# Supplementary material for: A recurrent sequencing artifact on Illumina sequencers with two-color fluorescent dye chemistry and its impact on somatic variant detection
Source: Genome Biol. 2026 Apr 17;27:179. doi: 10.1186/s13059-026-04081-3 (PMC13218037; doi:10.1186/s13059-026-04081-3)
Supplement: Supplementary file 2 — Additional file 2: Supplementary Figure 1. z-statistics after simulating pileup counts if the NovaSeq and HiSeq coverages were similar. Related to Figure 3. Supplementary Figure 2. Enrichment of T>G variant calls in NovaSeq 6000 vs HiSeq X10 for HG006 and HG007 where WGS datasets across platforms are downsampled to similar coverages. Supplementary Figure 3. Base composition surrounding T>A/A>T and T>C/A>G mutations in the six platform-unmatched deephuman cortical samples. Supplementary Figure 4. Comparisons of the proportions of low-allele depthT>A, T>C, and T>G calls across all samples analyzed in Figure 3. 99.9% CIs are shown. [file 13059_2026_4081_MOESM2_ESM.docx]

**Supplementary Material**

[Supplementary Figure 1 2](#_Toc221437651)

[Supplementary Figure 2 3](#_Toc221437652)

[Supplementary Figure 3 4](#_Toc221437653)

[Supplementary Figure 4 5](#_Toc221437654)

[Supplementary Information 1 6](#_Toc221437655)

[Supplementary Table 1 6](#_Toc221437656)

[Supplementary Table 2 6](#_Toc221437657)

[Supplementary Table 3 6](#_Toc221437658)

[Supplementary Table 4 6](#_Toc221437659)

# Supplementary Figure 1

z-statistics after simulating pileup counts if the NovaSeq and HiSeq coverages were similar. Related to Figure 3.

# Supplementary Figure 2

Enrichment of T>G variant calls in NovaSeq 6000 vs HiSeqX 10 for HG006 and HG007 where WGS datasets across platforms are downsampled to similar coverages. Results from downsampling analysis are shown in blue; the original track as presented in Figure 3A is shown in gold.

# Supplementary Figure 3

Base composition surrounding T>A/A>T and T>C/A>G mutations in the six platform-unmatched deep (>200X) human cortical samples (UMB4638, UMB4643, UMB5575, UMB5580; BA9, BA17, and BA18). The figure is laid out as in **Figure 4D.**

# Supplementary Figure 4

Comparisons of the proportions of low-allele depth (AD 2-5) T>A, T>C, and T>G calls across all samples analyzed in Figure 3. 99.9% CI’s are shown.

# Supplementary Information 1

Tables of the counts and proportions of all pileups of single-base substitutions (C>A, C>G, C>T, T>A, T>G, T>G) at different allele depths (AD) in the 14 different samples assessed for Figure 2. The difference in proportions between different platforms is given, along with a summary of proportions over different AD ranges for AD > 10.

# Supplementary Table 1

Metadata of 14 pairs of samples sequenced on both NovaSeq and HiSeq. The “Reference” column shows the reference genome to which the original dataset was aligned. For the variant-calling analysis, all samples were aligned to GRCh38. The “post-processing coverage” column indicates the coverage after applying the stringent read processing prior to substitution pileup analysis (**Figure 2**).

# Supplementary Table 2

List of filters applied during TNFilter to process mutation calls for the variant-level analysis (**Figure 3**).

# Supplementary Table 3

Metadata of deeply sequenced (>200X) human cortical samples. “Post-Procesing Coverage” refers to the BAM coverage after applying GATK4 Best Practices, not the read pileup pre-processing as in **Supplementary Table 1** or **Figure 2**.

# Supplementary Table 4

Summary of the k-mer (k=4) sequences found in the 20-base windows around different single-base substitutions discovered in the human cortical samples.
